# The Elements of Materia Medica and Therapeutics

**Published:** 1844-01

**Authors:** 


### REVIEWS.

---

Art. V.—*The Elements of Materia Medica and Therapeutics.* By Jonathan Pereira, M.D., F. R. S. & L. S., Licentiate of the Royal College of Physicians in London; Member of the Royal College of Surgeons; Fellow of the Royal Medical and Chirurgical Society; Corresponding Member of the Society of Pharmacy of Paris; Honorary Member of the Pharmaceutical Society of Great Britain; Examiner in Materia Medica and Pharmacy to the University of London; and Assistant Physician to, and Lecturer on Materia Medica at, the London Hospital. *With numerous illustrations.* From the Second London Edition, enlarged and improved. *With notes and additions.* By Joseph Carson, M.D., Professor of Materia Medica and Pharmacy in the Philadelphia College of Pharmacy, and one of the Editors of the American Journal of Pharmacy. In two vols., 8vo. pp. 714 and 852. Philadelphia. Lea & Blanchard. 1843.

"After a long and tedious discourse," says old Burton, in his Anatomy of Melancholy, "of these six non-natural things, and their several rectifications, all which are comprehended in diet, I am come now at last to *Pharmaceutice*, or that kind of physic which cureth by medicines, which apothecaries most part make, mingle or sell in their shops. Many cavil at this kind of physic, and hold it unnecessary, unprofitable to this or any other disease, because those countries which use it least, live longest and are best in health, as Hector Boëthius relates of the Isles of Orcades, the people are still sound of body and mind, without any use of physic; they live commonly one hundred and twenty years. Paulus Jovius, in his description of Britain, and Levinus Lemnius, observe as much of this island, that there was of old no use of physic amongst us, and but little at this day, except it be for a few nice, idle citizens, surfeiting courtiers, and stall-fed gentlemen lubbers. The country people use kitchen physic, and common experience tells us, that they live freest from all manner of infirmities, that make least use of apothecaries' physic."

Times are greatly changed in that wonderful island since the days of Paulus Jovius, and of the quaint author of the Anatomy of Melancholy, and men have changed with them. No doubt, now, as then, the "nice, idle citizens" take more than a fair proportion of the apothecary's physic, but the products of the culinary art no longer suffice for the happy inhabitants of the country. Hygeia has ceased to be the propitious divinity, lengthening the days of men to six score years, and the present short-lived race of mortals are doomed to seek succor from the disciples of Esculapius. It were indeed bad for our numerous "flourishing" medical schools, with their crowds of young aspirants after fees and fame, if the consumption of physic were not increasing, *pari passu*, with the growth of our population. The splendid work, the title of which stands at the head of this article, affords indubitable proof of the prosperous condition of the apothecary's art. Here we have nearly sixteen hundred closely printed pages on Materia Medica; not diluted—spun out to make a big book, but pages of condensed matter, the writer being evidently a man who has a just appreciation of the value of time. The author speaks of it as merely a faithful "outline" of this department of medicine, and considering the vast range of subjects embraced in it, it is surprising that the work should have been brought within such reasonable limits. Besides what belongs strictly to Materia Medica, it contains a concise account of the most important modern discoveries in Natural History, Chemistry, Physiology, and Therapeutics, in so far as they pertain to Pharmacology. It is a singularly complete work. We have heard it objected to, as being too learned for the medical pupils of our country; but, we confess, we like it all the better for its learning. The study necessary to master it will make the more accomplished practitioners. It exhibits the true connexions between pharmacy, chemistry, physiology, and therapeutics, and the reader of Pereira must have a pretty thorough acquaintance with these branches in order to read him with satisfaction. It is the very work, in our judgment, which the wants of the American profession demand. It will give a higher interest to the study of Materia Medica, and enhance its dignity as a branch of medical education, because it is exhibited in this work more fully than ever before, in any English treatise, in its philosophical bearings. Every practitioner ought to supply himself with a copy of it for his own use, and especially for the benefit of his pupils, the thorough study of which should make an indispensable part of their medical education.

Prefixed to the elements is a *Tabular view of the History and Literature of the Materia Medica*, beginning with Egyptian medicine, seventeen centuries before the christian era, extending over all countries where it has been cultivated, and coming down to the publication of Dr. Dunglison's Therapeutics and Materia Medica in the year just closed. This is a fair specimen of the character of Pereira's Materia Medica. It omits nothing. When a subject is barely introduced, such references are given by the author as will enable the curious reader to pursue his researches to the utmost extent of his wishes.

The first part of this work treats of General Therapeutics, including psychical, or mental remedies, as external and internal affections of the mind; and corporal remedies, as the imponderable agents, light, heat, electricity, and magnetism; hygienic agents, as food, exercise and climate; and pharmacological agents, or medicines, the means of ascertaining their effects, their active forces, their physiological effects, their absorption into the living system, their operation by nervous agency, the parts affected by the remote action of the medicines, the general nature of the effects of medicines, the modification of their effects by circumstances, their therapeutical effects, the parts to which they are applied, and their pharmacological classification. We shall not attempt to follow the learned author in the discussion of all these topics, much less to give an analysis of his matter, which would be impossible in a space much more limited than than that of the work itself, but shall select, here and there, such passages as may appear to us likely to interest our readers, and afford to them, at the same time, the means of judging of the value of these Elements.

Under the head of internal affections of the mind, our author has the following remarks:

"Emotions and passions of the mind have a most powerful influence upon the disorders of the body. Much of the evidence, however, which establishes the truth of this statement, is rather curious than practically useful, and as the general fact is well known and admitted, I shall confine myself to a few practical illustrations. *Hope* is a mildly stimulating or tonic passion, which may be beneficially employed in all diseases, and which proves injurious in few, if in any cases. Most patients receive with satisfaction and benefit, assurances of the prospect of recovery from their medical attendant. Even in diseases of a mortal charcter, life may be sometimes prolonged, by concealing from the sufferer the fatal nature of his malady. I fully agree with the learned President of the College of Physicians, Sir H. Halford, that the first duty of the physician is 'to protract the life of his patient by all practicable means.'"

We do not believe that the physician is required by any obligation to deceive his patients. It is unquestionably his duty to protract their lives to the utmost of his ability, but we can readily suppose that a few hours of painful life might be purchased at too high a price. It is the right of the patient to be informed of his true condition; he has claims upon his physician to deal with him in candor, as well as in gentleness; and we doubt whether a rev.elation of the truth to the sufferer, if done with tenderness and judgment, has ever given a fatal termination to disease, or materially shortened life. An amiable weakness of our nature inclines us to deceive the sick upon a question, to them, of the most momentous concern. It is better that we should resist the temptation, and act towards our patients as becomes christians and honest men. But it is no part of our purpose in this article to write a homily, and so we proceed to Materia Medica.

The vitiated air of cow-houses as a remedy in pulmonary diseases, we supposed had gone out of use since the time of Beddoes; but Dr. Pereira publishes a note from his pupil, Mr. Steinhausen, of Saxony, from which it would appear that this remedial agent still holds its ground in that country.

"In Germany," he says, "the balsamic air of cow-sheds is commonly recommended as a preventive in suspected pulmonary disease, or as a means of prolonging life in a confirmed phthisis. Although this latter disease is comparatively of rare occurrence in Saxony, yet several cases have fallen under my observation in which this plan of treatment was adopted. The mode of effecting it has varied according to circumstances: in some cases the patient has merely retired from a crowded town to a farm-house; in others, the sitting and bed-rooms have actually been converted into residences for cows. Of the former I have known several instances, where patients have been greatly benefited by sleeping in apartments built over cow-stalls; and this, I should say, is the most usual plan adopted."

The benefit derived from breathing this air by pulmonary invalids, our author thinks, is referrible in part to its warmth and moisture, but he supposes that the carbonic acid which is also present, may dilute the oxygen of the air and render it less stimulating and hurtful.

Cold, as a curative agent, is treated of with sufficient minuteness, but our author has not recommended it as highly as it deserves to be. The profession is not yet alive to the value of ice, cold drinks, and cold applications in our febrile and inflammatory affections. Many practitioners are to be met with who are still afraid to allow patients laboring under fever, cholera infantum, and similar complaints, the free use of ice. So benign have we found the action of cold in such cases, that we could wish every writer on Materia Medica to insist upon the application of cooling remedies wherever admissible, as among the greatest improvements in modern medicine.

Under the head of *voltaic electricity*, we find the following suggestion, which if not new to our readers, deserves to be quoted for its ingenuity:

"*To electrolyze urinary calculi.* Prévost and Dumas have proposed voltaic electricity as a means of destroying some kinds of urinary calculi. They state that a fusible calculus, in one case, contained in a basin of water, in another introduced into the bladder of a dog previously distended with water, was completely disintegrated by voltaic electricity, from a battery of 120 pairs. The wires were introduced through a canula into the bladder. The operation, it is said, did not occasion the least apparent uneasiness to the animal. During the action of the battery on the calculus, the bases and phosphoric acid first arrived at their respective poles, then re-entered into combination, forming a pulverulent salt. Bonnet proposed to inject the bladder with a solution of nitrate of potash, and to galvanize the calculus in this liquor. The nitrate will be decomposed; the phosphates dissolve in the liberated nitric acid,—and the uric acid, or urate of ammonia, in the disengaged potash. These propositions are ingenious, but at present no practical use has been made of them."

Of all forms of electrical apparatus, that which is most simple, convenient and powerful, is the magneto-electrical machine, devised by Mr. E. M. Clarke, of London, very perfect instruments of which kind are made in this city, by Mr. Jacob Walter. It is an instrument which deserves the attention of the profession. Interesting cures by its agency are reported in all the journals of the day, and we have ourselves witnessed its beneficial application in some cases of neuralgia. It has the advantage over all other electrical instruments of being independent of the weather, requiring no acid for its excitation, and being always in readiness for action. In suspended animation, whether by narcotic poisons, lightning, drowning, or the mephitic gases, its application would appear to have been successful under circumstances which would not have admitted of the employment of any other remedy.

We pass over the chapter on Food, as we propose to make the Treatise on Diet by the same author, the subject of an article in a future number. The chapter on Climate is one of great interest to the pulmonary invalid, but the reader must consult the work itself for full information on the subject.

The following remarks on the active forces of medicines have practical interest:

"Chemical changes are sometimes produced in the secretions of distant parts by the internal use of certain agents. Thus the qualities of the urine are modified by the administration of acids or alkalies. These modifications or changes depend, at least in a considerable number of instances, on the chemical influence of the substances swallowed. For when either acids or alkalis are swallowed, they pass out of the system, in part at least, by the kidneys, and in the urine they possess their usual chemical properties, modified by the presence of any substances with which they may have united. Moreover, the qualities which they impress on the urine, are similar to those which they produce when added to this secretion after its evacuation from the bladder. Thus, by the internal use of alkalis, it has been found that the natural acidity of the urine may be destroyed, and an alkaline quality substituted for it: the same condition of urine is produced by the addition of alkalis to this fluid out of the body. Again, the internal use of soda or magnesia may give rise to the appearance of white sand (phosphates) in the urine: and the same kind of deposite is produced in healthy urine by the addition of a few drops of an alkaline solution to it. Furthermore, by the administration of acids (sulphuric or hydrochloric) phosphatic deposits are diminished or entirely prevented, while the employment of alkalis promotes them: and a few drops of hydrochloric acid added to urine, in which the earthy phosphates are suspended, dissolve them. In other words, as the modifications which acids and alkalis produce in the condition of the urine, are precisely those which might be expected from the known chemical properties of these bodies, it is rational to infer that they depend on the affinities of these substances."

The absorption of medicines forms the subject of a highly interesting chapter, but as it is rather speculative than practical, we shall make no extracts from it, nor shall we stop to notice the chapters on the operation of medicines by nervous agency, and the circumstances which modify the effects of medicines, in which will be found a clear analysis of all that is known on these subjects. We must also pass, without comment, his remarks on pharmacological classification. He treats medicines in the order of their "natural-historical relations," dividing them into two groups, *the inorganized and the organized*, the latter being further divided into two sub-kingdoms, *the vegetable and the animal.*

Oxygen stands at the head of the first class. Soon after the discovery of this gas the most exaggerated notions prevailed as to its remedial powers. Our author supposes it may be useful in cases of asphyxia arising from the inhalation of noxious vapors. An instance of the resuscitation of a dog from drowning by inflating the lungs with oxygen, is recorded by Dr. Muse, in the American Journal of Science and Arts, vol. xvi., p. 250. In the same work, (vol. i., p. 95,) other interesting cases are detailed. On the whole, however, Pereira believes oxygen to be almost useless as a remedy. Of chlorine he has a better opinion. It is used advantageously as a wash in skin diseases, as a gargle in putrid sore throat; as a local bath in liver diseases, and as an application to ulcers attended with a fœtid discharge. Inhaled in chronic pulmonary diseases it is, according to his experience, essentially serviceable in ulceration of the lungs, although useless in tubercular phthisis for which it has been prescribed.

The article on *Iodine* is one which will be read by the practitioner with great satisfaction. This remedy is shown by numerous references to have effected striking cures in bronchocele, scrofula, chronic induration and enlargement of the liver, spleen, uterus, and prostate, parotid and lymphatic glands, gonorrhœa, leucorrhœa, the venereal disease, and many of the affections of the skin. In erysipelas, Pereira has found it highly beneficial. In checking or controlling the ulcerative process, Mr. Key proved it to be one of the most powerful remedies we possess, the most active phagedænic ulcers putting on a healthy granulating appearance under its influence. In phlegmonous inflammation, sloughing of the cellular membrane, inflammation of the absorbents, gout, carbuncle, whitlow, lacerated, contused, and punctured wounds, and burns, and scalds, authority is cited for its remedial powers. "Its topical uses are, therefore, nearly as extensive as those of nitrate of silver. Moreover, it is used in the same classes of cases, and with the same views." Its uses are well summed up by our author in the following paragraph:

"As a remedial agent iodine is principally valuable for its resolvent influence in chronic visceral and glandular enlargements, indurations, thickening of membranes (as of the periosteum), and in tumors. In comparing its therapeutical power with that of mercury, we observe in the first place that it is not adapted for febrile and acute inflammatory complaints, in several of which mercury proves a most valuable agent. Indeed the existence of inflammatory fever is a contra-indication for the employment of iodine. Secondly, iodine is especially adapted for scrofulous,—mercury for syphilitic, maladies; and it is well known that in the former class of diseases mercurials are for the most part injurious. Thirdly, the influence of iodine over the secreting organs is much less constant and powerful than that of mercury;—so that in retention or suppression of the secretions, mercury is for the most part greatly superior to iodine. Fourthly, iodine evinces a specific influence over the diseases of certain organs (*e. g.* the thyroid body), which mercury does not. These are some only of the peculiarities which distinguish the therapeutical action of iodine from that of mercury."

Under the head of *Water*, he gives the following account of the *Methodus Hydriatica*, or famous water cure, for the application of which he states thirteen or fourteen establishments have been formed in Germany within the last few years:

"The following is a sketch of the regimen usually adopted at these establishments:

"At four, or half past four in the morning, perspiration is begun to be produced, which is done by wrapping the patients, like babies, in swaddling clothes, or like mummies, in large and thick blankets. Perspiration usually begins in an hour, and is kept up by making the patients drink several glasses of cold water every half hour. In many cases, when the perspiration is at its height, pieces of cloth dipped in cold water, and previously well wrung, are dexterously introduced under the blankets, and applied to the most diseased parts; these parts, as well as the cloths, grow hot, the perspiration soon begins afresh, and causes a sensation of burning in the part.

"At the end of three hours the blankets and bed are soaked with perspiration; the patients are then conducted into a neighboring room, or to another story, where they take their cold baths; and in doing this they often pass through draughts of air without being inconvenienced. Before plunging into the cold bath, they wash their head and chest; and after staying in it two or three minutes, the patients take a few cups of milk with a little bread, and then walk out upon the mountains which border on the establishment, and drink cold water at the numerous springs which they meet upon their route. About nine or ten o'clock they take the douche, or else walk to the cascades which are in the forests or mountains, and expose themselves to the fall of the water that comes down from a height, and strikes the body with great force. Immediately after dressing, they again drink several glasses of water, and then walk in the open air. (*London Medical Gazette*, for October 12, 1839, p. 111).

"This mode of treatment is recommended for old and young, males and females, and is followed both in summer and winter. It is regarded as a kind of universal remedy or panacea. Thus Oertel (*Die allerneuesten Wasserkuren*, 18 Hefte, Nürnberg, 1829–37), says it is good for affections of the eyes, ears, and teeth, for insanity, epilepsy, hydrophobia, erysipelas, quinsy, bronchial phthisis, inflammation of the brain, chest, or abdomen, faintings, diseases of the liver and spleen, gout, stomach complaints, acute eruptions, hemorrhages, alvine obstructions, &c!"

The most ample information on all the various classes and remedial uses of water is afforded in the section on that subject, but we shall make but one more extract from this part of the work, which relates to a somewhat novel application of water—it is as follows:

"What is called *Water-dressing* may be regarded as a modified and improved form of poultice. It consists in the application of two or three layers of soft lint dipped in water and applied to inflamed parts, wounds, and ulcers;—the whole being covered with oiled silk or India rubber, which should project beyond the margin of the lint, to retain the moisture and prevent evaporation. Dr. Macartney (*Treatise on Inflammation*, p. 180 London, 1838), considers it to operate differently to a poultice: unlike the latter, he says, it prevents or diminishes the secretion of pus, checks the formation of exuberant granulations, and removes all pain: moreover, the water is not apt to become sour, like a poultice, and does not injure the sound part."

*Hydrochloric acid in dyspepsia.* "Two facts," says Pereira, "give a remarkable interest to the employment of this acid in dyspepsia—namely, that it is a constituent of the gastric juice; and, secondly, that when mingled with mucus it has the power of dissolving various articles of food." The fact of its beneficial action in some cases of indigestion is as well established, as that of its presence in the healthy stomach, or of its agency in the process of chymification. It is esteemed a good remedy against the phosphatic deposites in urine, and the generation of intestinal worms.

Of *Ammonia*, he says:

"If we compare the effects of ammonia with those of other stimulants, as camphor, wine and opium, we observe, in the first place, that the influence of ammonia is principally manifested in the ganglionic and true spinal systems,—while the other stimulants, above mentioned, affect the cerebral system. Thus the effects of ammonia are usually exhibited on the circulation, respiration, secretion, and spasmodic actions: but camphor, wine, and opium, though they also affect these functions, yet they principally affect the intellectual functions. Secondly, the effects of ammonia are more transient than those of the other agents just referred to. Thirdly, the vascular excitement caused by wine and opium is attended with diminished mucous secretion, and is allied more to an ordinary febrile attack."

The caution suggested as to its continued employment in dyspeptic complaints is judicious. Most practitioners, probably, have seen individuals laboring under dyspepsia injured by the long-continued use of alkalis.

"It is to be remembered," he remarks, "that the healthy secretions of the stomach are of an acid nature, and that the continued use of ammonia or any other alkali, must ultimately be attended with injurious results, more especially to the digestive functions. While, therefore, the occasional employment of alkalis may be serviceable, their constant or long-continued use must ultimately prove deleterious."

Employed as a vesicatory, our author claims for it two advantages over cantharides—a more speedy operation, and non-affection of the urinary organs. For this purpose the strongest aqua ammoniæ is used. In cases of bites of venomous reptiles and rabid animals, and stings of insects, it is a popular remedy. The relief afforded in the sting of the bee is instantaneous.

In connexion with the natural history of *carbonic acid*, the following curious account of the *Valley of Poison*, in Java, a gigantic *Grotto del Cane*, is given:

"It is a cavity of an oval form, about three-quarters of a mile in circumference, and from thirty to thirty-five feet deep; filled to the height of about eighteen feet with carbonic acid gas. The bottom of it is covered with the skeletons of men and various other animals, who have fallen victims to its destructive operation. If a traveller should be so unfortunate as to enter it, he cannot be sensible of his danger until too late to return. Mr. Loudon thrust a dog in: he fell in fourteen seconds. A fowl thrown in appeared to be dead before it reached the ground."

*Creasote* is an article which, although in use for a number of years past, may be classed among the *new remedies*.

"As an internal remedy," says our author, "creasote has been principally celebrated, in this country, as a remedy possessing extraordinary powers of arresting vomiting. It has, however, been greatly overrated. It is decidedly injurious in inflammatory conditions and structural disease of the stomach, and frequently fails in allaying the sickness dependent on organic diseases, as of the heart and kidneys. It is most successful in hysterical cases, and sometimes succeeds in pregnancy.

In gastrodynia or flatulence it occasionally succeeds, but is admissible in those cases only in which local stimulants are usually found beneficial. Where both creasote and hydrocyanic acid have been separately tried without success, Dr. Elliotson advises their union."

Its use in tooth-ache is familiar in this country, and externally it is further applied with benefit to foul and indolent ulcers, and in hemorrhages it is a good deal employed as a styptic. Creasote water is recommended in uterine hemorrhage, introduced into the vagina by means of pledgets of lint soaked in it, and as an application to bleeding wounds and leech-bites. Hemorrhage from the umbilical cord in infants is one of the cases in which creasote is an efficient remedy. We quote from our author other remedial uses of which it is susceptible:

"It has been employed to check caries, to restrain excessive suppuration, and to repress fungous granulations in burns and scalds; to act as a counter-irritant in chronic ophthalmia, in which disease it is sometimes dropped into the eye on the same principle that nitrate of silver and other local stimulants are used; and to remove condylomatous and other excrescences. The inhalation of creasote vapor is occasionally useful in relieving excessive bronchial secretions. Dr. Elliotson cured two cases of chronic glanders in the human subject by injecting an aqueous solution of creasote up the affected nostril."

Pereira cites cases of poisoning by creasote, but the symptoms usually yield readily to appropriate remedies. Mr. O., of this city, 61 years of age, and of infirm health, took a tea-spoonful of the article, which had been got a few days previously from an apothecary to be applied for toothache, on the 11th instant, mistaking it for a mixture of balsam copaivæ and spirits of nitre, which he was using for an irritable bladder. His wife, so soon as the error was discovered, gave him a table-spoonful of melted hog's lard. In a few minutes he took an emetic of ipecac. and tart. antimon., which operated freely. The only symptoms of which he complained were a burning in the stomach and a pain of the head. He used diluent drinks, and gentle purgatives, and was relieved from all the effects of the creasote on the third day, and at the same time found himself free from the irritability of the bladder, for which he had been using medicine. Whether the creasote had any agency in the cure of this affection is not clear.

Under the head of Sulphur, as a remedy in scabies, we find the following remark, from which it will be seen that the question as to the origin of this annoying disease in the irritation of the *itch-insect*, is not yet settled.

"But before adopting this explanation of the *modus medendi* of sulphur, it is to be proved that the animal is the *cause* of the disease; for, at present, it has not been satisfactorily shown whether it be the cause, effect, or mere accompaniment of itch. Rayer observes, that it is indubitable that the number of these insects bears no proportion to that of the vesicles. 'It is, farther,' he adds, 'rare to discover these insects on the abdomen and on groins, where the eruption of scabies is nevertheless very common and very apparent; moreover, scabies is known to continue when no more acari are to be discovered.'"

*Iodide of Potassium* is a remedy of which a very extensive use is now made by the practitioners of this country, as well as of Europe; it is therefore of importance that the mode of testing its purity should be generally understood. Our author says,

"Iodide of potassium is often largely adulterated with *carbonate of potash.* In 1829 I analyzed a sample, which contained 77 per cent. of the latter salt. In one specimen Dr. Christison procured 74.5 per cent. of carbonate of potash, 16 of water, and only 9.5 of iodide of potassium. The impure salt may be distinguished by its wanting any regular crystalline form; by adding a few particles of it to lime-water, a milky fluid is obtained, whereas the liquid remains transparent if the iodide be pure; by its destroying the color of the tincture of iodine, whereas the pure salt does not affect it; and lastly, by alcohol, which dissolves iodide of potassium, but not carbonate of potash."

"The following are the characters of pure iodide of potassium according to the *London College:*

"Totally soluble in water and in alcohol. It alters the color of turmeric either not at all or very slightly. It does not alter the color of litmus. Subjected to heat it loses no weight. Sulphuric acid and starch added together it becomes blue. Ten grains of this salt are sufficient to decompose 10.24 grains of nitrite of silver: what is precipitated is partly dissolved by nitric acid, and partly altered in appearance; which is not the case when ammonia is added."

To many of our readers the subjoined extract will probably be interesting—on the uses of hydriodate of potash:

"Having so fully detailed the uses of iodine, it is unnecessary to notice at any length those of iodide of potassium, since they are for the most part identical. Thus it has been employed in bronchocele, scrofula, in chronic diseases accompanied with induration and enlargement of various organs, in leucorrhœa, secondary syphilis, periostitis, articular rheumatism, dropsies, &c. As a remedy for the hard periosteal node brought on by syphilis, it was first employed by Dr. Williams, who obtained with it uniform success. At the end of from five to ten days its mitigating effects are felt; the pains are relieved, the node begins to subside, and in the majority of cases disappears altogether. In these cases, Dr. Clendinning has also borne testimony to its efficacy. In the tubercular forms of venereal eruptions, Dr. Williams found it beneficial. In Dr. Wallace's lectures are some valuable observations on the use of iodide of potassium in venereal diseases. In chronic rheumatism accompanied with alteration in the condition of the textures of the joint, it is, in some cases, remarkably successful. As an ingredient for baths, Lugol found the iodide would not answer alone, but that it was useful as a solvent means for iodine."

From the following it would appear that the *Herculean Calomel-practice*, as it has been styled, is not peculiar to our country, nor can the facts given in the quotation be regarded as very unfavorable to that practice in the disease specified:

"The largest quantity of calomel given as a medicinal agent, at one dose, is, I believe, three drachms; 'and it was followed,' says Dr. Christison, from whom I quote the case, which occurred in America, 'by only one copious evacuation, and that not till after the use of an injection.' I have now before me reports of eighteen cases of spasmodic cholera, admitted in the year 1832 into the Cholera Hospital at Bethnal Green, in this metropolis, in which enormous quantities of calomel were employed by the house-surgeon, Mr. Charles Bennett, with very slight physiological effects. When a patient was brought into the hospital, two drachms of calomel were immediately given, and afterwards one drachm every one or two hours, until some effect was produced. In 17 out of 18 cases in which this plan was tried, the vomiting and purging diminished, and the patients recovered. Several of them took from 20 to 30 drachms without the subsequent ptyalism being at all excessive. In one case, 30½ drachms were administered within forty-eight hours; moderate ptyalism took place, and recovery. In the unsuccessful case which I have alluded to, 53 drachms of calomel were administered within forty-two hours, without the least sensible effect."

The chapter on *Iron and its compounds* contains much valuable matter. Of iron, in its relation to that state of the system denominated *anæmia*, in which both the quantity and quality of the blood appear defective, the following remarks possess practical interest—they refer to *chlorosis:*

"If in this condition of system we administer iron, the appetite increases, digestion is promoted, the pulse becomes fuller and stronger, the skin assumes its natural tint, the lips and cheeks become more florid, the temperature of the body is increased, the œdema disappears, and the muscular strength is greatly augmented. The alvine evacuations assume a black color, as they always do under the use of the ferruginous preparations. After continuing the use of iron for a few weeks, we frequently find excitement of the vascular system (particularly the brain); thus we have throbbing of the cerebral vessels, and sometimes pain in the head, a febrile condition of system, with a tendency to hemorrhage. Mr. Carmichael considers the *sanguine* temperament (marked by a high complexion, celerity of thought, remarkable irritability of fibre, and a quick pulse) as depending on an excess of iron in the system; whereas the *leucophlegmatic*, or *relaxed*, temperament (characterized by a pale bloated countenance, dull eyes, mind heavy and slow in receiving and forming ideas, little irritability of fibre, and pulse small and feeble) as depending on a deficiency of iron.

"When by the use of iron the state of the general system improves, the secretions resume their natural condition; and thus at one time we observe this metal promoting the uterine discharge, at another checking it, according as chlorosis or menorrhagia had been previously present; we cannot, therefore, regard the preparations of this metal as having any direct emmenagogue effect, as some have supposed.

"Some refer all the other symptoms of anæmia to the abnormal state of the blood, and ascribe the beneficial influence of iron to the improvement in the quality of this liquid. It is certain that, under the use of the preparations of this metal, the blood frequently acquires a more scarlet color, owing probably to an increase in the number of its coloring particles; and the crassamentum becomes firmer and more solid, and even increased in quantity. This alteration of the physical and chemical properties of the blood must render it more stimulating, and thus the different organs, receiving a fluid of a more healthy character, resume their normal condition, and perform their functions in a proper manner. Tiedmann and Gmelin have detected it in the serum of the blood of the portal and mesenteric veins of horses and dogs, to whom they administered either the sulphate or chloride. Occasionally, too, iron has been found in the urine. Moreover, Menghini asserts, that the quantity of iron in the blood of dogs may be increased by feeding them on substances mixed with this metal. Furthermore, it is not to be forgotten, that iron exists in no inconsiderable quantity in healthy blood, and is supposed to contribute to its color, and probably to its stimulant properties; so that it is not unlikely any variation in the quantity of this metal would be attended with an alteration in the action of every organ."

The second, and larger volume is devoted to the history of medicines derived from the *organized kingdom.* The details given are so clear and ample, that the practitioner will not often have to seek for further information concerning any of the articles mentioned. As in the description of mineral substanes, all that relates to their natural history, chemical constitution, physiological effects, and remedial uses, is concisely, but so fully related, that it would be difficult to add any thing of value to the descriptions of the author, without going into details of practice inadmissible in an elementary work. Equally difficult, perhaps, would it be to find omitted in these volumes, any single remedial agent of established value. Many of very equivocal claims to any healing virtues are admitted into the catalogue; many are mentioned which are decidedly worthless; and those which the practitioner will probably never employ are introduced by hundreds; but such must be the character of every treatise which assumes to be a complete history of Materia Medica.

The following extract from the article on *Ergot* will afford an idea of the minuteness with which the author details all that is known about a medicinal substance:

"The nature and formation of ergot are subjects on which botanists have been much divided in opinion.

"Some regard ergot as a fungus growing between the glumes of grasses in the place of the ovary. Otto Von Münchausen, Schrank, De Candolle, Fries, Wiggers, and Berkeley, have adopted this opinion, and have described ergot as a fungus under the name of *Spermoedia Clavus.* Fries and Berkeley, however, evidently entertain some doubts respecting its nature; for the first adds to the generic character of Spermoedia, "*Semina graminum morbosa,*" and the second says, "it appears to be only a diseased state of the grain, and has scarcely a sufficient claim to be admitted among fungi as a distinct genus."

"Against this opinion may be urged the circumstance noticed by Tessier, that a part only of the grain may be ergotized. Moreover, the scales of the base of the ergot, the frequent remains of the stigma on its top, and the articulation of it to the receptacle, prove that it is not an independent fungus, but an altered grain.

"Some regard ergot as a diseased condition of the ovary or seed. The arguments adduced against the last opinion are in favor of the present one. Though a considerable number of writers have taken this view of the nature of ergot, there has been great discordance among them as to the causes which produced the disease.

"*Some have supposed that ordinary morbific causes, as moisture combined with warmth, were sufficient to give rise to this diseased condition of the grain.* Tessier and Wildenow appear to have been of this opinion.

"*Some have ascribed the disease to the attack of insects or other animals.* Tillet, Fontana, Read, and Field, supported this view, which, I may add, has subsequently been satisfactorily disproved.

"*Some, dissatisfied with the previonsly assigned causes of the disease, have been content with declaring ergot to be a disease, but without specifying the circumstances which induce it.* Mr. Bauer, who closely watched the development of ergot during eight years, and has made some beautiful drawings of it in different stages, arrived at this conclusion; as also Phœbus.

"*Others have referred the disease to a parasitic fungus.* This opinion, which must not be confounded with that entertained by De Candolle and others, has been adopted and supported by Léveille, in 1826 by Dutrochet, Smith, and by Quekett."

The remedial applications of this article are stated at much length. We make room for a few quotations:

"Another interesting topic of inquiry is the action of ergot on the gravid uterus of mammals. Chapman says 'it never fails, in a short time, to occasion abortion.' We have the testimony of Percy and Laurent, that a decoction injected into the veins of a cow caused the animal to calve speedily; and in one out of three experiments, Mr. Combes has stated, the ergot caused the abortion of a bitch, Diez found that it caused uterine contractions in dogs, rabbits, and sows. Large doses given to bitches induced an inflammatory condition of the uterus, and destroyed both mother and her young. However, in opposition to these statements, we have the evidence of Chatard, Warner, Villeneuve, and others, who failed in producing abortion with it.

"I am indebted to Mr. Youatt, Veterinary Surgeon to the Zoological Society, and editor of the *Veterinarian*, for the following note respecting the effects of ergot on animals:

"'I have, for the last six or seven years, been in the habit of administering the ergot of rye to quadrupeds in cases of difficult or protracted parturition, in order to stimulate the uterus to renewed or increased action. In the *monogastric*, if I may venture to use the term, I have never known it fail of producing considerable effect, even when the uterus had been previously exhausted by continued and violent efforts. In the *ruminant*, with its compound stomach or stomcahs, I have witnessed many a case of its successful exhibition. I have had recourse to it in the cow, the sheep, and the deer, both foreign and domestic. Parturition has not always been accomplished, from false presentation or other causes, but the uterus has in every case responded—it has been roused to a greater or less degree of renewed reaction. On the other hand, there are cases recorded by veterinary practitioners, in which it has been given in very large quantities without producing the slightest effect. I have always attributed this to a certain degree of forgetfulness of the construction of the stomachs of ruminants. If the medicine, as is too often the case, is poured hastily down, and from a large vessel, it breaks through the floor of the œsophagean canal and falls into the rumen, and there it remains perfectly inert. But if it is suffered to trickle down the œsophagean canal, although a portion of it may still enter the rumen, the greater part will flow on through the œsophagean canal and the manyplies into the fourth or villous stomach, and produce the desired effect.'"

"*Effects on the uterine system.* The action of spurred rye on the uterus *when labour has actually commenced*, is usually observed in from ten to twenty minutes after the medicine has been taken, and is manifested by an increase in the violence, the continuance, and the frequency of the pains, which usually never cease until the child is born; nay they often continue for some minutes after, and promote the speedy separation of the placenta and the firm contraction of the uterus in a globular form. The contractions and pains caused by ergot are distinguished from those of natural labour by their continuance; scarcely any interval can be perceived between them, but a sensation is experienced of one continued forcing effort. If from any mechanical impediment (as distortion) the uterus cannot get rid of its contents, the violence of its contraction may cause its rupture, as in the cases alluded to by Dr. Merriman, Mr. Armstrong, and Mr. Coward.

"Ergot sometimes fails to excite uterine contractions. The causes of failure are for the most part conjectural. The quality of the ergot, peculiarities on the part of the mother, and death of the fœtus, have been assigned as such. The two first will be readily admitted; but why the remedy should be altogether inert "where the fœtus has been for sometime dead, and putrefaction to any extent taken place" cannot be readily explained. Its occasional failure has been urged by Dr. Hamilton as an argument in favor of his notion that ergot acts "in no other way than by influencing the imagination." But on the same ground the sialogogue power of mercury might be denied. Dr. Hamilton's erroneous estimate of the powers of ergot is referrible to a want of experience of its use; for he admits that he has only had two opportunities in practice of making a fair trial of it.

"There is usually much less hemorrhage after delivery, when ergot has been employed, than where it has not been exhibited. The lochial discharges are also said to be less; but this is certainly not constantly the case. Moreover, it has been asserted 'that the menstrual discharge has not recurred after the use of the ergot in certain cases of protracted parturition.' But the inference intended to be conveyed here, viz. that ergot caused the non-recurrence, is not correct; at least, I am acquainted with several cases in which this effect did not follow the employment of spurred rye, and I know of none in which it did.

"Ergot has been charged with causing the death of the child; but the charge has been repelled by some experienced practitioners as being devoid of the least foundation. 'The ergot,' says Dr. Hosack, 'has been called in some of the books, from its effects in hastening labour, the *pulvis ad partum;* as it regards the child, it may with almost equal truth be denominated the *pulvis ad mortem*, for I believe its operation, when sufficient to expel the child, in cases alone where nature alone is unequal to the task, is to produce so violent a contraction of the womb, and consequent convolution and compression of the uterine vessels, as very much to impede, if not totally to interrupt the circulation between mother and child.' However, Dr. Chapman strongly denies this charge, and tells us that in 200 cases which occurred in tne practice of himself and Drs. Dewees and James, the ergot was used without doing harm in any respect; and he adds, 'no one here believes in the alleged deleterious influence of the article on the fœtus.' It is not improbable, however, where the impediment to labour is very great, that the violent action of the uterus may be attended with the result stated by Dr. Hosack. Dr. F. H. Ramsbotham has suggested that the poisonous influence of ergot may be extended from the mother to the fœtus, as in the case of opium. He also states that of 36 cases in which he induced premature labour by puncturing the membranes, 21 children were born alive; while in 26 cases of premature labour induced by ergot only, 12 children only were born alive. This fact strongly favors the notion of the deleterious influence of ergot on the fœtus.

"Given to excite abortion, or premature labour, ergot has sometimes failed to produce the desired effect. Hence many experienced accoucheurs have concluded, that for this medicine to have any effect on the uterus it was necessary that the process of labour should have actually commenced. But while we admit that it sometimes fails, we have abundant evidence to prove that it frequently succeeds; and most practitioners, I think, are now satisfied that, in a large number of cases, it has the power of originating the process of accouchement.

"*To restrian uteterine hemorrhage, whether puerperal or non-puerperal.*—Ergot checks hemorrhage from the womb, principally, if not solely, by exciting contraction of the muscular fibres of this viscus, by which its blood-vessels are compressed and emptied, and their orifices closed. The experience of physicians and surgeons in all parts of the civilized world has fully and incontestibly established the efficacy of ergot as a remedy for uterine hemorrhage. Maisonneuve and Trosseau, have shown that the beneficial influence of ergot is exerted equally in the unimpregnated as in the impregnated state; proving, therefore, that the contrary statement of Prescott and Villeneuve is incorrect. Even in a case of cancer of the uterus they have found it check the sanguineous discharge. In females subject to profuse uterine hemorrhages after delivery, ergot may be administered as a preventive, just before the birth of the child. Even in placental presentations, a dose or two of ergot may be administered previously to the delivery being undertaken. To restrain excessive discharge of the lochia or catamenia, this remedy is sometimes most beneficial.

"*In hemorrhages generally.*—The power possessed by ergot of exciting uterine contractions, readily explains the efficacy of this agent in restraining sanguineous discharges from the womb; but we can in no way understand how hemorrhage from other organs can be influenced by it. We are not, however, to deny the therapeutic power of a medicine merely because we cannot explain its *modus medendi*, though we are justified in requiring abundant proofs ere we admit it. It must be acknowledged, that a considerable number of cases have been published in proof of the power possessed by ergot of checking hemorrhages from other organs (as the nose, gums, chest, stomach, and rectum) than the uterus. But having found it unsuccessful in my own practice, seeing that in the hands of others it has also failed, and knowing how difficult it is to ascertain the influence of remedies on hemorrhages, I think further evidence is required to prove the anti-hemorrhagic powers of ergot."

We shall not multiply our extracts from this elaborate work, of the value of which we are fully aware that no just estimate can be formed from quotations. It is a production which to be appreciated must be seen and examined. That it will command, in this region, as in all other places where it has found its way, it has won, the approbation of the profession, may safely be predicted, and its extensive circulation will be at once an indication and an assurance of an increasing taste for thorough medical knowledge. Such a work cannot be made current without creating a desire for more enlarged information on all the branches of science connected with medicine—a desire which, to a very great extent, it is capable of gratifying. The American editor has added to it whatever was wanting to make it a complete description of the Materia Medica of the United States, and the publishers have got it up in good style. The illustrations are numerous and satisfactory. The table of contents, prepared, we are told, by the wife of the author, is not the least of the recommendations of a work embracing so vast a range of subjects. The table is so full that the reader will find every article and every topic of discussion mentioned in its place. We cannot doubt that the publishers will be amply rewarded for their enterprise, in bringing out a work of so much excellence. Y.

